
JOURNAL INFORMATION
==============================
NLM Title Abbreviation: Natl Sci Rev
Journal ID: Natl Sci Rev
NLM Journal ID: 101633095
Journal ID: nsr

National Science Review

ISSN: 2095-5138
EISSN: 2053-714X

ARTICLE INFORMATION
==============================
PMCID: PMC7717373
PMID: 34676093
DOI: 10.1093/nsr/nwaa285
Article ID: nwaa285
Article version: 1
Subjects: Brcomm, AcademicSubjects/MED00010, AcademicSubjects/SCI00010

Rapid generation of ACE2 humanized inbred mouse model for COVID-19 with tetraploid complementation

Liu Feng-Liang Key Laboratory of Animal Models and Human Disease Mechanisms of the Chinese Academy of Sciences/Key Laboratory of Bioactive Peptides of Yunnan Province, KIZ-CUHK Joint Laboratory of Bioresources and Molecular Research in Common Diseases, Kunming Institute of Zoology, Chinese Academy of Sciences, Kunming, Yunnan 650223, China

Wu Kaixin Center for Cell Fate and Lineage (CCLA), Bioland Laboratory (Guangzhou Regenerative Medicine and Health Guangdong Laboratory), 510005 Guangzhou, China
CAS Key Laboratory of Regenerative Biology, Guangdong Provincial Key Laboratory of Stem Cell and Regenerative Medicine, Guangzhou Institutes of Biomedicine and Health, Chinese Academy of Sciences, Guangzhou 510530, China
Department of Developmental Biology, School of Basic Medical Sciences, Southern Medical University, Guangzhou, 510515, China
Central Laboratory, The Sixth Affiliated Hospital of Guangzhou Medical University, Qingyuan People's Hospital, B24 Yinquan South Road, Qingyuan 511518, Guang Dong Province, China

Sun Jiaoyang CAS Key Laboratory of Regenerative Biology, Guangdong Provincial Key Laboratory of Stem Cell and Regenerative Medicine, Guangzhou Institutes of Biomedicine and Health, Chinese Academy of Sciences, Guangzhou 510530, China
University of the Chinese Academy of Sciences, Beijing 101408, China
Joint School of Life Science, Guangzhou Medical University, Guangzhou, China

Duan Zilei Key Laboratory of Animal Models and Human Disease Mechanisms of the Chinese Academy of Sciences/Key Laboratory of Bioactive Peptides of Yunnan Province, KIZ-CUHK Joint Laboratory of Bioresources and Molecular Research in Common Diseases, Kunming Institute of Zoology, Chinese Academy of Sciences, Kunming, Yunnan 650223, China

Quan Xiongzhi Center for Cell Fate and Lineage (CCLA), Bioland Laboratory (Guangzhou Regenerative Medicine and Health Guangdong Laboratory), 510005 Guangzhou, China
CAS Key Laboratory of Regenerative Biology, Guangdong Provincial Key Laboratory of Stem Cell and Regenerative Medicine, Guangzhou Institutes of Biomedicine and Health, Chinese Academy of Sciences, Guangzhou 510530, China

Kuang Junqi Center for Cell Fate and Lineage (CCLA), Bioland Laboratory (Guangzhou Regenerative Medicine and Health Guangdong Laboratory), 510005 Guangzhou, China
CAS Key Laboratory of Regenerative Biology, Guangdong Provincial Key Laboratory of Stem Cell and Regenerative Medicine, Guangzhou Institutes of Biomedicine and Health, Chinese Academy of Sciences, Guangzhou 510530, China
University of the Chinese Academy of Sciences, Beijing 101408, China

Chu Shilong Center for Cell Fate and Lineage (CCLA), Bioland Laboratory (Guangzhou Regenerative Medicine and Health Guangdong Laboratory), 510005 Guangzhou, China
CAS Key Laboratory of Regenerative Biology, Guangdong Provincial Key Laboratory of Stem Cell and Regenerative Medicine, Guangzhou Institutes of Biomedicine and Health, Chinese Academy of Sciences, Guangzhou 510530, China

Pang Wei Center for Cell Fate and Lineage (CCLA), Bioland Laboratory (Guangzhou Regenerative Medicine and Health Guangdong Laboratory), 510005 Guangzhou, China
CAS Key Laboratory of Regenerative Biology, Guangdong Provincial Key Laboratory of Stem Cell and Regenerative Medicine, Guangzhou Institutes of Biomedicine and Health, Chinese Academy of Sciences, Guangzhou 510530, China

Gao Han Center for Cell Fate and Lineage (CCLA), Bioland Laboratory (Guangzhou Regenerative Medicine and Health Guangdong Laboratory), 510005 Guangzhou, China

Xu Ling Key Laboratory of Animal Models and Human Disease Mechanisms of the Chinese Academy of Sciences/Key Laboratory of Bioactive Peptides of Yunnan Province, KIZ-CUHK Joint Laboratory of Bioresources and Molecular Research in Common Diseases, Kunming Institute of Zoology, Chinese Academy of Sciences, Kunming, Yunnan 650223, China

Li Ying-Chang Key Laboratory of Animal Models and Human Disease Mechanisms of the Chinese Academy of Sciences/Key Laboratory of Bioactive Peptides of Yunnan Province, KIZ-CUHK Joint Laboratory of Bioresources and Molecular Research in Common Diseases, Kunming Institute of Zoology, Chinese Academy of Sciences, Kunming, Yunnan 650223, China

Zhang Hai-Lin Key Laboratory of Animal Models and Human Disease Mechanisms of the Chinese Academy of Sciences/Key Laboratory of Bioactive Peptides of Yunnan Province, KIZ-CUHK Joint Laboratory of Bioresources and Molecular Research in Common Diseases, Kunming Institute of Zoology, Chinese Academy of Sciences, Kunming, Yunnan 650223, China

Wang Xue-Hui Key Laboratory of Animal Models and Human Disease Mechanisms of the Chinese Academy of Sciences/Key Laboratory of Bioactive Peptides of Yunnan Province, KIZ-CUHK Joint Laboratory of Bioresources and Molecular Research in Common Diseases, Kunming Institute of Zoology, Chinese Academy of Sciences, Kunming, Yunnan 650223, China

Luo Rong-Hua Key Laboratory of Animal Models and Human Disease Mechanisms of the Chinese Academy of Sciences/Key Laboratory of Bioactive Peptides of Yunnan Province, KIZ-CUHK Joint Laboratory of Bioresources and Molecular Research in Common Diseases, Kunming Institute of Zoology, Chinese Academy of Sciences, Kunming, Yunnan 650223, China

Feng Xiao-Li Kunming National High-level Biosafety Research Center for Non-human Primates, Center for Biosafety Mega-Science, Kunming Institute of Zoology Chinese Academic of Sciences, Kunming, Yunnan 650107, China

Schöler Hans R Department of Cell and Developmental Biology, Max Planck Institute for Molecular Biomedicine, Röntgenstrasse 20, Münster 48149, Germany
Medical Faculty, University of Münster, Domagkstrasse 3, Münster 48149, Germany

Chen Xinwen CAS Key Laboratory of Regenerative Biology, Guangdong Provincial Key Laboratory of Stem Cell and Regenerative Medicine, Guangzhou Institutes of Biomedicine and Health, Chinese Academy of Sciences, Guangzhou 510530, China
University of the Chinese Academy of Sciences, Beijing 101408, China

Pei Duanqing Center for Cell Fate and Lineage (CCLA), Bioland Laboratory (Guangzhou Regenerative Medicine and Health Guangdong Laboratory), 510005 Guangzhou, China

Wu Guangming wu_guangming@grmh-gdl.cn Center for Cell Fate and Lineage (CCLA), Bioland Laboratory (Guangzhou Regenerative Medicine and Health Guangdong Laboratory), 510005 Guangzhou, China

Zheng Yong-Tang zhengyt@mail.kiz.ac.cn Key Laboratory of Animal Models and Human Disease Mechanisms of the Chinese Academy of Sciences/Key Laboratory of Bioactive Peptides of Yunnan Province, KIZ-CUHK Joint Laboratory of Bioresources and Molecular Research in Common Diseases, Kunming Institute of Zoology, Chinese Academy of Sciences, Kunming, Yunnan 650223, China
Kunming National High-level Biosafety Research Center for Non-human Primates, Center for Biosafety Mega-Science, Kunming Institute of Zoology Chinese Academic of Sciences, Kunming, Yunnan 650107, China

Chen Jiekai chen_jiekai@gibh.ac.cn Center for Cell Fate and Lineage (CCLA), Bioland Laboratory (Guangzhou Regenerative Medicine and Health Guangdong Laboratory), 510005 Guangzhou, China
CAS Key Laboratory of Regenerative Biology, Guangdong Provincial Key Laboratory of Stem Cell and Regenerative Medicine, Guangzhou Institutes of Biomedicine and Health, Chinese Academy of Sciences, Guangzhou 510530, China
Joint School of Life Science, Guangzhou Medical University, Guangzhou, China

Corresponding author. E-mail: wu_guangming@grmh-gdl.cnCorresponding author. E-mail: zhengyt@mail.kiz.ac.cnCorresponding author. E-mail: chen_jiekai@gibh.ac.cnThese authors contributed equally to this work.

Electronic publication date: 2020 Nov 24
Electronic Location ID: nwaa285
Copyright: © The Author(s) 2020. Published by Oxford University Press on behalf of China Science Publishing & Media Ltd.
Copyright year: 2020
License: This is an Open Access article distributed under the terms of the Creative Commons Attribution License (http://creativecommons.org/licenses/by/4.0/), which permits unrestricted reuse, distribution, and reproduction in any medium, provided the original work is properly cited.
License URL: https://creativecommons.org/licenses/by/4.0/

edited-state: accepted-manuscript
article-lifecycle: PAP

==============================
BRIEF COMMUNICATION

MOLECULAR BIOLOGY & GENETICS

### Rapid generation of ACE2 humanized inbred mouse model for COVID-19 with tetraploid complementation

Feng-Liang Liu[1#], Kaixin Wu[2, 3, 7, 8 #], Jiaoyang Sun[3, 4, 6#], Zilei Duan[1#], Xiongzhi Quan[2, 3], Junqi Kuang[2, 3, 4], Shilong Chu[2, 3,], Wei Pang[2, 3], Han Gao[2], Ling Xu[1], Ying-Chang Li[1], Hai-Lin Zhang[1], Xue-Hui Wang[1], Rong-Hua Luo[1], Xiao-Li Feng[5], Hans R. Schöler[9,10], Xinwen Chen[3, 4], Duanqing Pei[2], Guangming Wu[2*], Yong-Tang Zheng[1,5*], Jiekai Chen[2, 3, 6, *]

1 Key Laboratory of Animal Models and Human Disease Mechanisms of the Chinese Academy of Sciences/Key Laboratory of Bioactive Peptides of Yunnan Province, KIZ-CUHK Joint Laboratory of Bioresources and Molecular Research in Common Diseases, Kunming Institute of Zoology, Chinese Academy of Sciences, Kunming, Yunnan 650223, China.

2 Center for Cell Fate and Lineage (CCLA), Bioland Laboratory (Guangzhou Regenerative Medicine and Health Guangdong Laboratory), 510005 Guangzhou, China

3 CAS Key Laboratory of Regenerative Biology, Guangdong Provincial Key Laboratory of Stem Cell and Regenerative Medicine, Guangzhou Institutes of Biomedicine and Health, Chinese Academy of Sciences, Guangzhou 510530, China.

4 University of the Chinese Academy of Sciences, Beijing 101408, China.

5 Kunming National High-level Biosafety Research Center for Non-human Primates, Center for Biosafety Mega-Science, Kunming Institute of Zoology Chinese Academic of Sciences, Kunming, Yunnan 650107, China.

6 Joint School of Life Science, Guangzhou Medical University, Guangzhou, China

7 Department of Developmental Biology, School of Basic Medical Sciences, Southern Medical University, Guangzhou, 510515, China

8 Central Laboratory, The Sixth Affiliated Hospital of Guangzhou Medical University, Qingyuan People's Hospital, B24 Yinquan South Road, Qingyuan 511518, Guang Dong Province, China

ORIGINAL UNEDITED MANUSCRIPT

© The Author(s) 2020. Published by Oxford University Press on behalf of China Science Publishing & Media Ltd. This is an Open Access article distributed under the terms of the Creative Commons Attribution License (http://creativecommons.org/licenses/by/4.0/), which permits unrestricted reuse, distribution, and reproduction in any medium, provided the original work is properly cited.

9 Department of Cell and Developmental Biology, Max Planck Institute for Molecular Biomedicine, Röntgenstrasse 20, Münster 48149, Germany
10 Medical Faculty, University of Münster, Domagkstrasse 3, Münster 48149, Germany

### These authors contributed equally to this work
* Correspondence
Corresponding: Prof. Guangming Wu (wu_guangming@grmh-gdl.cn); Prof. Yong-Tang Zheng (zhengyt@mail.kiz.ac.cn); Prof. Jiekai Chen (chen_jiekai@gibh.ac.cn).

ORIGINAL UNEDITED MANUSCRIPT

Although monkeys and pigs can be infected with SARS-CoV-2, they generally have a long growth cycle and a large size that is difficult to handle in large quantities. However, the readily available rats and mice are not susceptible to SARS-CoV-2 due to the difference in ACE2 (angiotensin-converting enzyme 2), the receptor mediating cell-entry of SARS-CoV-2(1). Thus, the key to establishing a mouse model of COVID-19 is to make the mice to express human ACE2 protein and therefore become SARS-CoV-2 susceptible.

However, the recent reported COVID-19 mouse models using random insertion transgene approach (2, 3) or adenoviral approach to express hACE2 (4), lack the tissue-specificity of hACE2 expression and might not replicate COVID-19 precisely, limiting their application in certain studies. Therefore, precise genetic editing to express hACE2 specifically as endogenous pattern is imperative. Mouse embryonic stem cells (ESCs) have the capacity to generate all ESC-derived mice through tetraploid complementation (Fig. S1A). It is possible to generate humanized mice much faster by combining tetraploid complementation with the efficient CRISPR/Cas9 mediated genome editing, as the traditional chimeric or pronuclear injection procedures need germline transmission step and usually takes at least half a year (Fig. S1B). But in the past, the tetraploid complementation assay only worked well with selected F1 hybrid ESCs, and failed to produce live pups with inbred background ESCs (5). In this study, we screened our newly derived ESC lines from the 2 commonly used inbred strains of mice, C57BL/6 and BALB/c, for high tetraploid complementation efficiency. By combining tetraploid complementation with the selected ESCs edited by CRISPR/Cas9 mediated knock-in, we successfully generated the ACE2 humanized inbred mice within 35 days (Tables S1-2), termed C57BL/6N-Ace2$^{em2(hACE2\text{-}WPRE,pgk\text{-}puro)/Gdl}$ and BALB/c-Ace2$^{em1(hACE2\text{-}WPRE,pgk\text{-}puro)/Gdl}$ (Figs. 1A and B), and abbreviated as B6 hACE2 and BALB/c hACE2 mice. We even generated the ACE2 humanized mice with female BALB/c ES lines, so we are able to expand the colony quickly by reproduction. Our data demonstrated that the humanized ACE2 mice expressed hACE2 specifically in the lung, kidney, testis, and intestine (Figs. 1C-E and Figs. S1AC-D).

To determine whether the ACE2 humanized mice could support SARS-CoV-2 replication and whether the virus challenged mice would show any symptoms as observed in human patients, we first intranasally infected the B6 hACE2 mice with SARS-CoV-2 at a dose of $2\times10^{6}$ $TCID_{50}$, and then sacrificed the mice at the indicated days post-infection (dpi) to collect samples, including lung and airway (Fig. S2A). We found that SARS-CoV-2 challenge resulted in body weight loss but no other visible clinical symptoms such as fever (Figs. S2B, S2C). Next, we measured the virus distribution among mouse tissues using qRT-PCR. As a result, the virus could be detected in all mice's respiratory system including lungs and airways (100% SARS-CoV-$2^{+}$), and the viral quantities were the highest at 1 and 3 dpi among all time points post-infection at the level of over $10^{6}$ and $10^{4}$ copies/μg tissue total RNA, respectively (Fig. 1F) as well as the most SARS-CoV-$2^{+}$ cells in the lung located in the respiratory epithelium (Fig. S2D). Further, the virus was also detected in the small intestine (Fig. 1F). Thus our data proved that our hACE2 mice could support SARS-CoV-2 replication. Many recent studies have reported that SARS-CoV-2 RNA can be detected in stool and anal swaps in the clinic (6, 7), proposing a possible fecal-mediated virus transmission route. Similar to the human patients, high level of SARS-CoV-2 was detected in the hACE2 mouse feces (Fig. 1F), indicating that the ACE2 humanized mice could be an ideal model to study the fecal transmission of SARS-CoV-2. In addition, it is hard to detect viral RNA in the kidney: SRAS-CoV-2 was only detected in one of seven mice at 1 dpi (data not shown). This data suggests that SARS-CoV-2 can infect the kidney but the load is very low, consistent with the observation in human (8).

Histological assay of lung showed serious lung injury in the middle of the infection progression, while the injury was mild at the beginning and the end of infection (Fig. S3N). Alveolar septal thickening, hemorrhage, and mild inflammatory cell infiltration were observed at most days post-infection (dpi) (Figs. S3B-M). At 5 dpi when the most serious lung injury appeared, two mice showed consolidation and hyaline membrane formation in the lung as evidence of ARDS (Figs. S3E and S3H). Taking together, the pathological data suggest that SARS-CoV-2 infection in hACE2 mice led to acute lung damage and ARDS as observed in clinical patients (9).

Advantage of this technology is the suitability with different genetic backgrounds. The generation of both B6 and BALB/c hACE2 mice allows us to compare the effect of genetic background on SARS-CoV-2. Similar to B6 hACE2 mice, SARS-CoV-2 was detected in the lung, airway, and small intestines of BALB/c hACE2 mice, but less virus was detected in BALB/c hACE2 mice compared with B6 hACE2 mice (Figs. S4A-C). Lung injuries including alveolar septal thickening, hemorrhage, inflammatory cell infiltration, and hyaline membrane formation were also observed in BALB/c hACE2 mice, but the severest injury happened earlier than that in the B6 hACE2 mice (Figs. S4D-J).

Finally, to test whether the mouse model could be used in the evaluation of anti-SARS-CoV-2 treatment, we treated the B6 hACE2 mice before virus challenge with a SARS-CoV-2 neutralizing antibody, which was kindly provided by Jinghua Yan and has been demonstrated for its capability to suppress SARS-CoV-2 replication in vivo (10). Viral loading measurement showed that the neutralizing antibody treatment significantly suppress virus replication in lung and airway (Fig. S5), suggesting that our hACE2 mouse model could be an ideal tool for studying pathogenesis of COVID-19 and evaluating anti-SARS-CoV-2 treatment and vaccine.

In conclusion, we rapidly generated the ACE2 humanized mice of different genetic backgrounds in large scale without the reproduction step by combining tetraploid complementation and CRISPR/Cas9 procedures to meet the urgent need in the current pandemics of SARS-CoV-2. The ACE2 humanized mice overcame the natural resistance of mice to the SARS-CoV-2 infection and the virally infected mice manifested part of clinical symptoms as observed in the human patients. As a proof-of-principle, our efficient technology can be used to generate severe COVID-19 models over the hACE2 mice for the development of treatment against the severe COVID-19 and also quickly establish other mouse models in responding to other emergency situations in the future.

### ACKNOWLEDGE

We thank Dr. Changwen Ke (Guangdong Provincial Center for Disease Control and Prevention) for provided SARS-CoV-2 strain, Ms. Qi Geng (primate GLP safety assessment center of the Kunming Institute of Zoology) for technical help with the histological analysis. We are grateful of platform for cell lineage technology of Bioland laboratory, and Guangzhou Branch of the Supercomputing Center of Chinese Academy of Sciences for its support. This work was supported by the emergency grants for prevention and control of SARS-CoV-2 of Ministry of Science and Technology (2020YFC0842400) and Science and Technology Planning Project of Guangdong Province, China (2020B111108001, 2020B1212060052), Key Research & Development Program of Bioland Laboratory (Guangzhou Regenerative Medicine and Health Guangdong Laboratory) (2018GZR110104003), the National Key Research and Development Program of China (2019YFA0110200), the National Natural Science Foundation of China (31970879 and 32070794 and 32000503)，Frontier Science Research Program of the CAS (ZDBS-LY-SM007).

### AUTHOR CONTRIBUTIONS

J.K.C., Y.T.Z., and G.M.W. conceived, designed and supervised the project. F.L.L., K.X.W., J.Y.S., Z.L.D., X.Z.Q., J.Q.K., S.L.C., W.P., H.G., L.X., Y.C.L., X.H.W., R.H.L. and X.L.F. performed experiments. F.L.L., K.X.W. and H.L.Z analyzed data. F.L.L. and K.X.W. wrote the manuscript. Y.T.Z., J.K.C., and G.M.W. revised the manuscript. All authors read and approved the manuscript.

### COMPETING INTERESTS

The authors declare no competing interests.

### REFERENCE

1. Hoffmann, M, Kleine-Weber, H, Schroeder, S*, et al.* SARS-CoV-2 Cell Entry Depends on ACE2 and TMPRSS2 and Is Blocked by a Clinically Proven Protease Inhibitor. *Cell*. 2020; **181**(2): 271-80 e8.
2. Bao, L, Deng, W, Huang, B*, et al.* The pathogenicity of SARS-CoV-2 in hACE2 transgenic mice. *Nature*. 2020; **583**(7818): 830-3.
3. Jiang, RD, Liu, MQ, Chen, Y*, et al.* Pathogenesis of SARS-CoV-2 in Transgenic Mice Expressing Human Angiotensin-Converting Enzyme 2. *Cell*. 2020; **182**(1): 50-8 e8.
4. Sun, J, Zhuang, Z, Zheng, J*, et al.* Generation of a Broadly Useful Model for COVID-19 Pathogenesis, Vaccination, and Treatment. *Cell*. 2020; **182**(3): 734-43 e5.
5. Kirchain, SM, Hayward, AM, Mkandawire, JM*, et al.* Comparison of tetraploid blastocyst microinjection of outbred Crl:CD1(ICR), hybrid B6D2F1/Tac, and inbred C57BL/6NTac embryos for generation of mice derived from embryonic stem cells. *Comp Med*. 2008; **58**(2): 145-50.
6. Zhang, J, Wang, S, Xue, Y. Fecal specimen diagnosis 2019 novel coronavirus-infected pneumonia. *J Med Virol*. 2020; **92**(6): 680-2.
7. Xiao, F, Sun, J, Xu, Y*, et al.* Infectious SARS-CoV-2 in Feces of Patient with Severe COVID-19. *Emerg Infect Dis*. 2020; **26**(8): 1920-2.
8. Puelles, VG, Lutgehetmann, M, Lindenmeyer, MT*, et al.* Multiorgan and Renal Tropism of SARS-CoV-2. *N Engl J Med*. 2020; **383**(6): 590-2.
9. Xu, Z, Shi, L, Wang, Y*, et al.* Pathological findings of COVID-19 associated with acute respiratory distress syndrome. *Lancet Respir Med*. 2020; **8**(4): 420-2.
10. Shi, R, Shan, C, Duan, X*, et al.* A human neutralizing antibody targets the receptor binding site of SARS-CoV-2. *Nature*. 2020.

ORIGINAL UNEDITED MANUSCRIPT

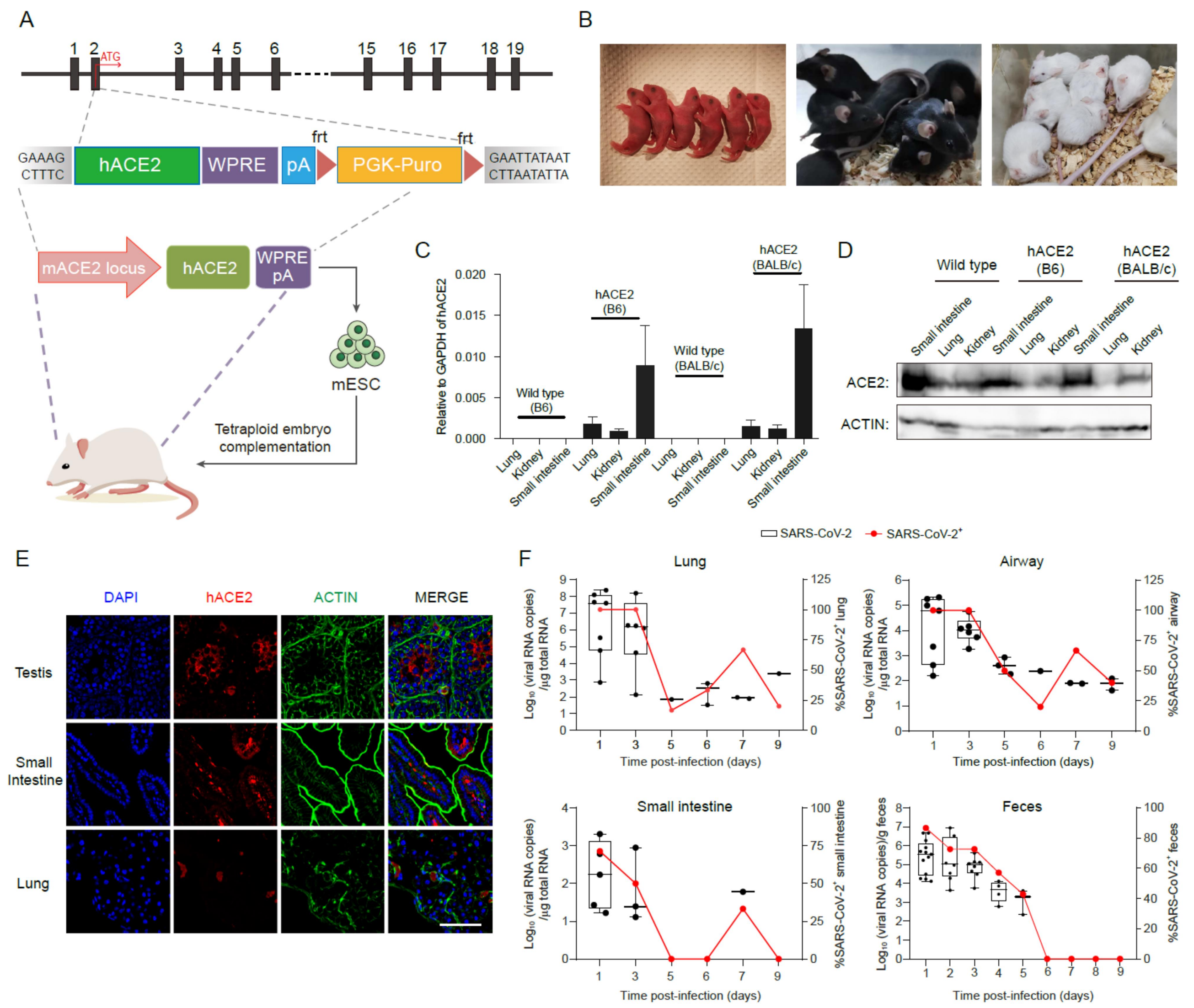

**Fig.1 Generation and characterization of ACE2 humanized inbred mouse model for COVID-19 with tetraploid complementation.**

(A) Strategy to generate ACE2 humanized mice via CRISPR-Cas9 system combined with tetraploid complementation. The hACE2 gene was inserted into exon 2, the first coding exon of mouse Ace2.

(B) Birth of hACE2 mice with the tetraploid complementation approach.

(C) The expression pattern of hACE2 in hACE2 mice (n=3) and wildtype mice (n=1) detected by RT-qPCR. Data are mean ± SD.

ORIGINAL UN

(D) Western blotting results of small intestine of hACE2 in humanized mice and wildtype mice. The primary antibody used here reacted with both mACE2 and hACE2 (ET1611-58, Huabio).

(E) Testis, small intestine and lung of hACE2 mice were stained for hACE2 (red), ACTIN filaments (green) and DAPI (blue). Scale bars, 50 μm.

(F) Lung, airway, small intestine and Fecal samples were collected after mice sacrifice at the indicated days post-infection. Total RNA was extracted and subjected to qRT-PCR for viral loading assay. The left y-axis showed the viral loading of each tissue or per gram feces, while the right y-axis showed the percentage of tissues containing SARS-CoV-2.

ORIGINAL UNEDITED MANUSCRIPT
