# Rapid generation of ACE2 humanized inbred mouse model for COVID-19 with tetraploid complementation

**DOI:** 10.1093/nsr/nwaa285

**Published:** 2020-11-24

**Authors:** Feng-Liang Liu, Kaixin Wu, Jiaoyang Sun, Zilei Duan, Xiongzhi Quan, Junqi Kuang, Shilong Chu, Wei Pang, Han Gao, Ling Xu, Ying-Chang Li, Hai-Lin Zhang, Xue-Hui Wang, Rong-Hua Luo, Xiao-Li Feng, Hans R Schöler, Xinwen Chen, Duanqing Pei, Guangming Wu, Yong-Tang Zheng, Jiekai Chen

**Affiliations:** Key Laboratory of Animal Models and Human Disease Mechanisms of the Chinese Academy of Sciences/Key Laboratory of Bioactive Peptides of Yunnan Province, KIZ-CUHK Joint Laboratory of Bioresources and Molecular Research in Common Diseases, Kunming Institute of Zoology, Chinese Academy of Sciences, China; Center for Cell Fate and Lineage (CCLA), Bioland Laboratory (Guangzhou Regenerative Medicine and Health Guangdong Laboratory), China; CAS Key Laboratory of Regenerative Biology, Guangdong Provincial Key Laboratory of Stem Cell and Regenerative Medicine, Guangzhou Institutes of Biomedicine and Health, Chinese Academy of Sciences, China; Department of Developmental Biology, School of Basic Medical Sciences, Southern Medical University, China; Central Laboratory, The Sixth Affiliated Hospital of Guangzhou Medical University, Qingyuan People's Hospital, China; CAS Key Laboratory of Regenerative Biology, Guangdong Provincial Key Laboratory of Stem Cell and Regenerative Medicine, Guangzhou Institutes of Biomedicine and Health, Chinese Academy of Sciences, China; University of the Chinese Academy of Sciences, China; Joint School of Life Science, Guangzhou Medical University, China; Key Laboratory of Animal Models and Human Disease Mechanisms of the Chinese Academy of Sciences/Key Laboratory of Bioactive Peptides of Yunnan Province, KIZ-CUHK Joint Laboratory of Bioresources and Molecular Research in Common Diseases, Kunming Institute of Zoology, Chinese Academy of Sciences, China; Center for Cell Fate and Lineage (CCLA), Bioland Laboratory (Guangzhou Regenerative Medicine and Health Guangdong Laboratory), China; CAS Key Laboratory of Regenerative Biology, Guangdong Provincial Key Laboratory of Stem Cell and Regenerative Medicine, Guangzhou Institutes of Biomedicine and Health, Chinese Academy of Sciences, China; Center for Cell Fate and Lineage (CCLA), Bioland Laboratory (Guangzhou Regenerative Medicine and Health Guangdong Laboratory), China; CAS Key Laboratory of Regenerative Biology, Guangdong Provincial Key Laboratory of Stem Cell and Regenerative Medicine, Guangzhou Institutes of Biomedicine and Health, Chinese Academy of Sciences, China; University of the Chinese Academy of Sciences, China; Center for Cell Fate and Lineage (CCLA), Bioland Laboratory (Guangzhou Regenerative Medicine and Health Guangdong Laboratory), China; CAS Key Laboratory of Regenerative Biology, Guangdong Provincial Key Laboratory of Stem Cell and Regenerative Medicine, Guangzhou Institutes of Biomedicine and Health, Chinese Academy of Sciences, China; Center for Cell Fate and Lineage (CCLA), Bioland Laboratory (Guangzhou Regenerative Medicine and Health Guangdong Laboratory), China; CAS Key Laboratory of Regenerative Biology, Guangdong Provincial Key Laboratory of Stem Cell and Regenerative Medicine, Guangzhou Institutes of Biomedicine and Health, Chinese Academy of Sciences, China; Center for Cell Fate and Lineage (CCLA), Bioland Laboratory (Guangzhou Regenerative Medicine and Health Guangdong Laboratory), China; Key Laboratory of Animal Models and Human Disease Mechanisms of the Chinese Academy of Sciences/Key Laboratory of Bioactive Peptides of Yunnan Province, KIZ-CUHK Joint Laboratory of Bioresources and Molecular Research in Common Diseases, Kunming Institute of Zoology, Chinese Academy of Sciences, China; Key Laboratory of Animal Models and Human Disease Mechanisms of the Chinese Academy of Sciences/Key Laboratory of Bioactive Peptides of Yunnan Province, KIZ-CUHK Joint Laboratory of Bioresources and Molecular Research in Common Diseases, Kunming Institute of Zoology, Chinese Academy of Sciences, China; Key Laboratory of Animal Models and Human Disease Mechanisms of the Chinese Academy of Sciences/Key Laboratory of Bioactive Peptides of Yunnan Province, KIZ-CUHK Joint Laboratory of Bioresources and Molecular Research in Common Diseases, Kunming Institute of Zoology, Chinese Academy of Sciences, China; Key Laboratory of Animal Models and Human Disease Mechanisms of the Chinese Academy of Sciences/Key Laboratory of Bioactive Peptides of Yunnan Province, KIZ-CUHK Joint Laboratory of Bioresources and Molecular Research in Common Diseases, Kunming Institute of Zoology, Chinese Academy of Sciences, China; Key Laboratory of Animal Models and Human Disease Mechanisms of the Chinese Academy of Sciences/Key Laboratory of Bioactive Peptides of Yunnan Province, KIZ-CUHK Joint Laboratory of Bioresources and Molecular Research in Common Diseases, Kunming Institute of Zoology, Chinese Academy of Sciences, China; Kunming National High-level Biosafety Research Center for Non-human Primates, Center for Biosafety Mega-Science, Kunming Institute of Zoology Chinese Academic of Sciences, China; Department of Cell and Developmental Biology, Max Planck Institute for Molecular Biomedicine, Germany; Medical Faculty, University of Münster, Germany; CAS Key Laboratory of Regenerative Biology, Guangdong Provincial Key Laboratory of Stem Cell and Regenerative Medicine, Guangzhou Institutes of Biomedicine and Health, Chinese Academy of Sciences, China; University of the Chinese Academy of Sciences, China; Center for Cell Fate and Lineage (CCLA), Bioland Laboratory (Guangzhou Regenerative Medicine and Health Guangdong Laboratory), China; Center for Cell Fate and Lineage (CCLA), Bioland Laboratory (Guangzhou Regenerative Medicine and Health Guangdong Laboratory), China; Key Laboratory of Animal Models and Human Disease Mechanisms of the Chinese Academy of Sciences/Key Laboratory of Bioactive Peptides of Yunnan Province, KIZ-CUHK Joint Laboratory of Bioresources and Molecular Research in Common Diseases, Kunming Institute of Zoology, Chinese Academy of Sciences, China; Center for Cell Fate and Lineage (CCLA), Bioland Laboratory (Guangzhou Regenerative Medicine and Health Guangdong Laboratory), China; Kunming National High-level Biosafety Research Center for Non-human Primates, Center for Biosafety Mega-Science, Kunming Institute of Zoology Chinese Academic of Sciences, China; Center for Cell Fate and Lineage (CCLA), Bioland Laboratory (Guangzhou Regenerative Medicine and Health Guangdong Laboratory), China; CAS Key Laboratory of Regenerative Biology, Guangdong Provincial Key Laboratory of Stem Cell and Regenerative Medicine, Guangzhou Institutes of Biomedicine and Health, Chinese Academy of Sciences, China; Joint School of Life Science, Guangzhou Medical University, China

Although monkeys and pigs can be infected with SARS-CoV-2, they generally have a long growth cycle and a large size that is difficult to handle in large quantities. However, the readily available rats and mice are not susceptible to SARS-CoV-2 because of differences in ACE2 (angiotensin-converting enzyme 2), the receptor mediating cell entry of SARS-CoV-2 [1]. Thus, the key to establishing a mouse model of COVID-19 is to make the mice express human ACE2 protein, and therefore become SARS-CoV-2 susceptible.

Recently reported COVID-19 mouse models using a random insertion transgene approach [2,3] or adenoviral approach to express hACE2 [4], lack the tissue-specificity of hACE2 expression and might not replicate COVID-19 precisely, limiting their applications in certain studies. Therefore, precise genetic editing to express hACE2 specifically as an endogenous pattern is imperative. Mouse embryonic stem cells (ESCs) have the capacity to generate all ESC-derived mice through tetraploid complementation (Fig. S1A). It is possible to generate humanized mice much faster by combining tetraploid complementation with efficient CRISPR/Cas9 mediated genome editing, as traditional chimeric or pronuclear injection procedures need a germline transmission step

and usually take at least half a year (Fig. S1B). In the past, the tetraploid complementation assay only worked well with selected F1 hybrid ESCs, and failed to produce live pups with inbred background ESCs [5]. In this study, we screened newly derived ESC lines from two commonly used inbred strains of mice, C57BL/6 and BALB/c, for high tetraploid complementation efficiency. By combining tetraploid complementation with the selected ESCs edited by CRISPR/Cas9 mediated knock-in, we successfully generated ACE2 humanized inbred mice within 35 days (Tables S1 and S2), termed C57BL/6N-Ace2^em2(hACE2-WPRE, pgk-puro)/CCLA^ and BALB/c-Ace2^em1(hACE2-WPRE,pgk-puro)/CCLA^ (Fig. 1A and B), and abbreviated as B6 hACE2 and BALB/c hACE2 mice. We even generated ACE2 humanized mice with female BALB/c ES lines, so we are able to expand the colony quickly by reproduction. Our data demonstrate that the humanized ACE2 mice expressed hACE2 specifically in the lung, kidney, testis and intestine (Fig. 1C–E and Fig. S1C and D).

**Figure 1.**
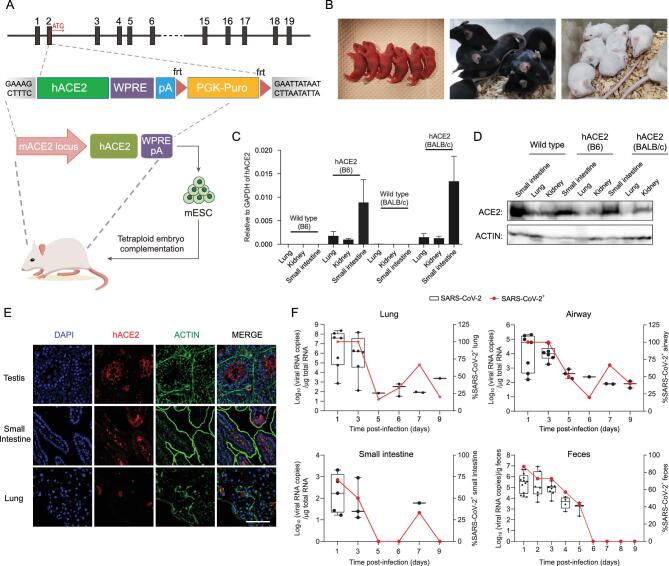
Generation and characterization of an ACE2 humanized inbred mouse model for COVID-19 with tetraploid complementation. (A) Strategy to generate ACE2 humanized mice via CRISPR-Cas9 system combined with tetraploid complementation. The hACE2 gene was inserted into exon 2, the first coding exon of mouse ACE2. (B) Birth of hACE2 mice with the tetraploid complementation approach. (C) Expression pattern of hACE2 in hACE2 mice (*n* = 3) and wildtype mice (*n* = 1) detected by RT-qPCR. Data are mean ± SD. (D) Western blotting results of small intestine, lung and kidney of hACE2 in humanized mice and wildtype mice. The primary antibody used here reacted with both mACE2 and hACE2 (ET1611–58, Huabio). (E) Testis, small intestine and lung of hACE2 mice were stained for hACE2 (red), ACTIN filaments (green) and DAPI (blue). Scale bars, 50 μm. (F) Lung, airway, small intestine and fecal samples were collected after mice sacrifice at the indicated days post-infection. Total RNA was extracted and subjected to qRT-PCR for viral loading assay. The left y-axis showed the viral loading of each tissue or per gram feces, while the right y-axis showed the percentage of tissues containing SARS-CoV-2.

To determine whether the ACE2 humanized mice could support SARS-CoV-2 replication and whether the virus challenged mice would show any symptoms as observed in human patients, we first intranasally infected

the B6 hACE2 mice with SARS-CoV-2 at a dose of 2 × 10^6^ TCID_50_, and then sacrificed the mice at the indicated days post-infection (dpi) to collect samples, including samples from the lung and airway (Fig. S2A). We found that SARS-CoV-2 challenge resulted in body weight loss but no other visible clinical symptoms such as fever (Fig. S2B and C). Next, we measured the virus distribution among mouse tissues using qRT-PCR. The virus was detected in the entire respiratory system of the mice, including lungs and airways (100% SARS-CoV-2^+^), with viral quantities highest at 1 and 3 dpi among all time points post-infection at levels of over 10^6^ and 10^4^ copies/μg tissue total RNA, respectively (Fig. 1F). The most SARS-CoV-2^+^ cells in the lung were located in the respiratory epithelium (Fig. S2D). The virus was also detected in the small intestine (Fig. 1F). These data prove that our hACE2 mice can support SARS-CoV-2 replication. Many recent studies have reported that SARS-CoV-2 RNA can be detected in stool and anal swabs in the clinic [6,7], proposing a possible fecal-mediated virus transmission route. Similar to human patients, high levels of SARS-CoV-2 were detected in feces from the hACE2 mice (Fig. 1F), indicating that the ACE2 humanized mice could be an ideal model to study

the fecal transmission of SARS-CoV-2. In addition, it is hard to detect viral RNA in the kidney: SRAS-CoV-2 was only detected in one of seven mice at 1 dpi (data not shown). These data suggest that SARS-CoV-2 can infect the kidney but the load is very low, consistent with observations in humans [8].

Histological assay of lung showed serious lung injury in the middle of the infection progression, while the injury was mild at the beginning and end of the infection (Fig. S3N). While alveolar septal thickening, hemorrhage and mild inflammatory cell infiltration were observed after infection (Fig. S3B-M), at 5 dpi the most serious lung injury appeared, two mice showed consolidation and hyaline membrane formation in the lung as evidence of acute respiratory distress syndrome (ARDS) (Fig. S3E and H). Taken together, the pathological data suggest

that SARS-CoV-2 infection in hACE2 mice led to acute lung damage and ARDS as observed in clinical patients [9].

One of the advantages of our animal model technology is suitability with different genetic backgrounds. Generation of both B6 and BALB/c hACE2 mice allows us to compare the effects of genetic background on SARS-CoV-2. Similar to B6 hACE2 mice, SARS-CoV-2 was detected in the lung, airway and small intestines of BALB/c hACE2 mice, but less virus was detected in BALB/c hACE2 mice compared with B6 hACE2 mice (Fig. S4A–C). Lung injuries, including alveolar septal thickening, hemorrhage, inflammatory cell infiltration and hyaline membrane formation, were also observed in BALB/c hACE2 mice, but the severest injury happened earlier than that in the B6 hACE2 mice (Fig. S4D–J).

Finally, to test whether the mouse model could be used in evaluation of anti-SARS-CoV-2 treatment, we treated the B6 hACE2 mice before virus challenge with a SARS-CoV-2 neutralizing antibody, which was kindly provided by Jinghua Yan and has been demonstrated to have the capability to suppress SARS-CoV-2 replication *in vivo* [10]. Viral loading measurement showed that the neutralizing antibody treatment significantly suppressed virus replication in the lung and airway (Fig. S5A and B), suggesting that our hACE2 mouse model could be an ideal tool for studying pathogenesis of COVID-19 and evaluating anti-SARS-CoV-2 treatment and vaccine.

In conclusion, we rapidly generated ACE2 humanized mice of different genetic backgrounds at large scale without a reproduction step, by combining tetraploid complementation and CRISPR/Cas9 procedures to meet the urgent need for the current pandemic of SARS-CoV-2. The ACE2 humanized mice overcame the natural resistance of mice to the SARS-CoV-2 infection and the virally infected mice manifested some of the clinical symptoms observed in human patients. As a proof-of-principle, our efficient technology can be used to generate animal models of severe disease based on the hACE2 mice for development of treatment against severe COVID-19 and also quickly establish specific mouse models in response to other emergency situations in the future.

## Supplementary Material

nwaa285_Supplement_File

## Notes

***Conflict of* *interest* *s* *tatement* *.*** None declared.
