# Supplementary material for: Rapid generation of ACE2 humanized inbred mouse model for COVID-19 with tetraploid complementation
Source: Natl Sci Rev. 2020 Nov 24;8(2):nwaa285. doi: 10.1093/nsr/nwaa285 (PMC7717373; doi:10.1093/nsr/nwaa285)
Supplement: nwaa285_Supplement_File [file nwaa285_supplement_file.zip › Fig_S1_S5.pdf]

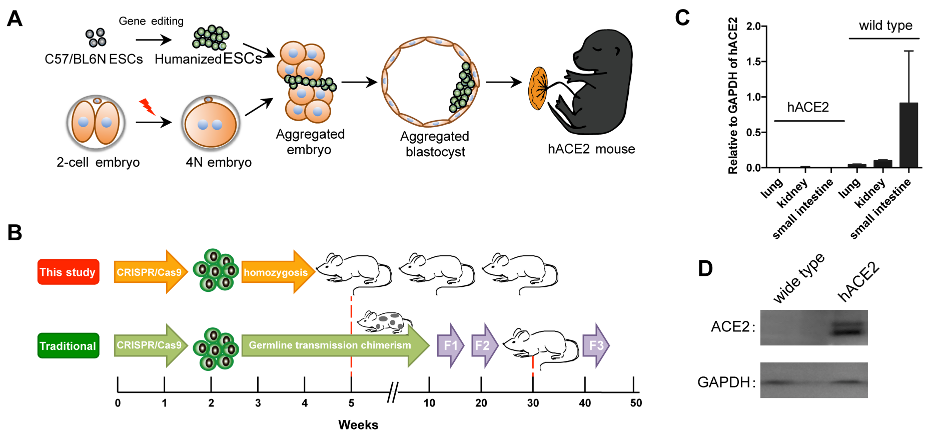

Fig. S1 Related to Fig. 1

(A) Schematic diagram of tetraploid compensation procedure.

(B) Schematic diagram of time comparison between traditional methods and tetraploid technology to generate hACE2 mice.

(C) The expression pattern of mACE2 in hACE2 mice and wildtype mice (n=3) detected by RT-qPCR.

(D) Western blotting results of small intestine of hACE2 in humanized mice and wildtype mice. The primary antibody used here only reacted with hACE2 (ab108209, Abcam).

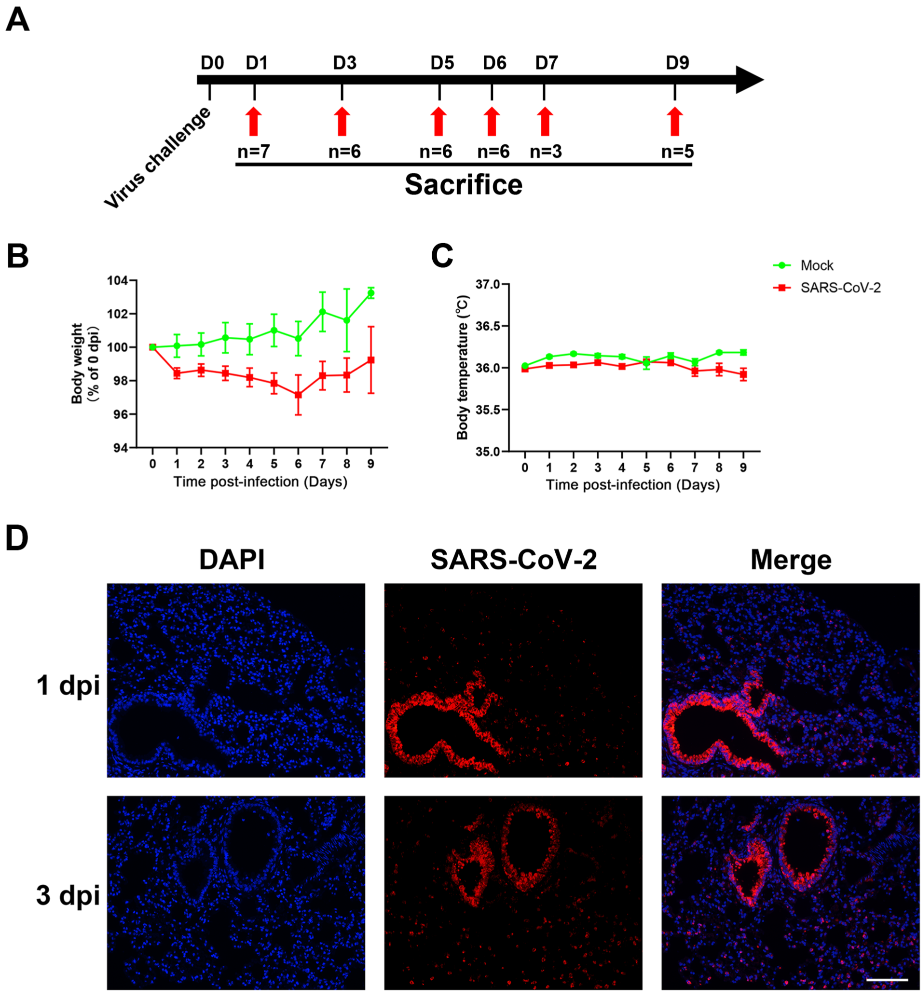

Fig. S2 The change of body weight and body temperature after virus challenge

(A) Experimental schedule for virus challenge. B6 hACE2 mice were infected intranasally with 2×106 TCID50 SARS-CoV-2, and were sacrificed at the indicated time for sample collection. n means the sacrificed mouse number at the indicated days post-infection.

(B and C) Body weight change and body temperature change post-infection. The body weight (B) and body temperature (C) of all mice including the mock control mouse were measured daily. Compared with the mock control, the SARS-CoV-2-infected mice showed a mild decrease of body weight after the virus infection. However, there was no obvious difference in body temperature between the virally infected mice and the mock control. Data are mean ± SEM. (D) SARS-CoV-2 Nucleocapsid protein was stained in the lung at 1 and 3 dpi, respectively. The sections were stained with anti-SARS-CoV-2 Nucleocapsid antibody (red) and DAPI (blue). Scale bar, 100μm.

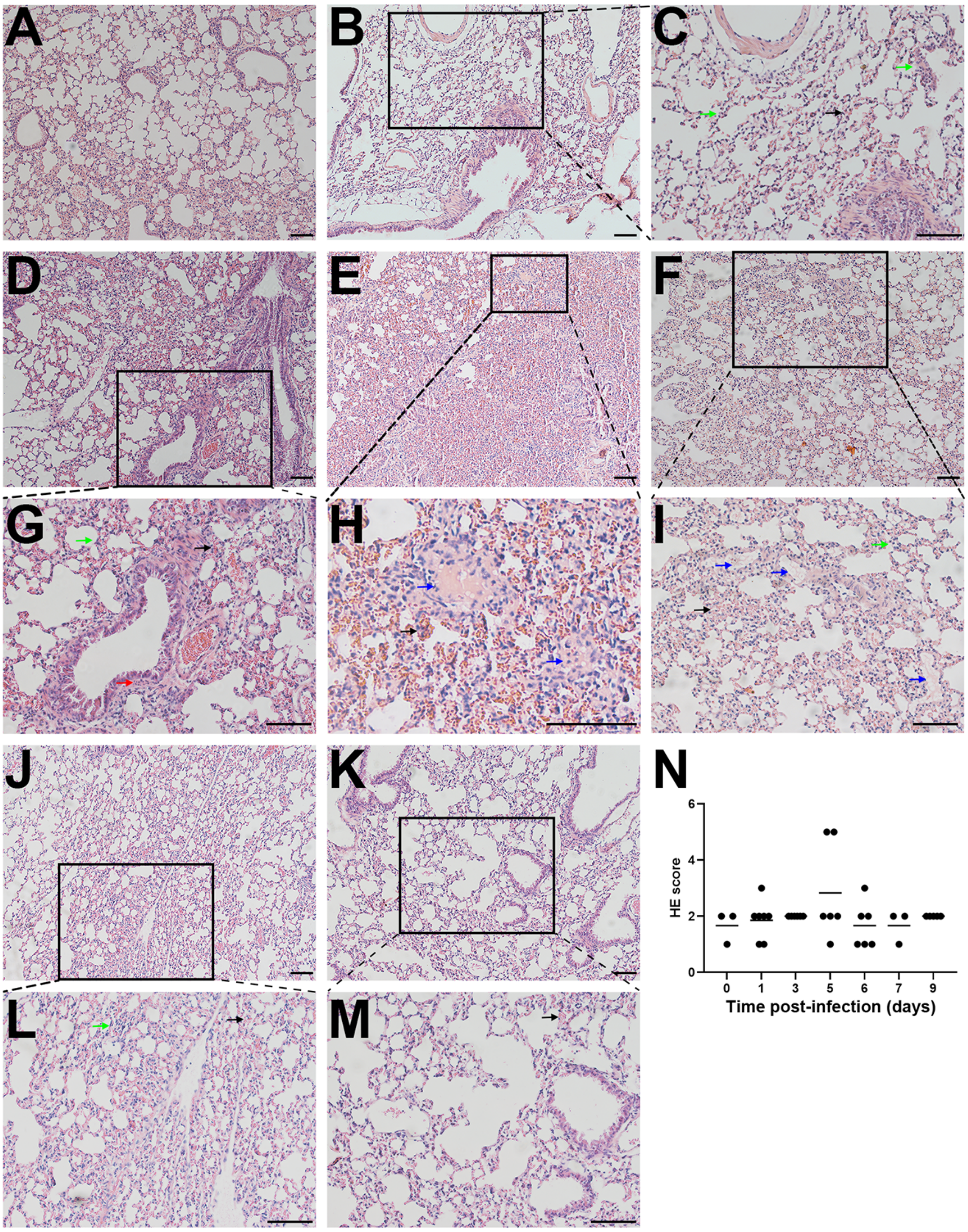

Fig. S3 Pathological assay in lung tissues of B6 hACE2 mice post-infection

Three mock control mice and all SARS-CoV-2-infected mice were used to assess the lung injury at the indicated time points post-infection.

(A) The mock control mice show no obvious lesion.

(B and C) Alveolar septal thickening (green arrow) and hemorrhage (black arrow) were observed in lung at 1 dpi.

(D and G) Alveolar septal thickening (green arrow), hemorrhage (black arrow), and inflammatory cell infiltration (red arrow) were observed in lung at 3 dpi.

(E and H) Consolidation, hyaline membrane formation (blue arrow), and hemorrhage (black arrow) were observed in lung at 5 dpi.

(F and I) Hemorrhage (black arrow) and alveolar septal thickening (green arrow) were observed in lung at 6 dpi. The hyaline membrane (blue arrow) was partly dissolved.

(J-M) Hemorrhage (black arrow) was observed in lung at 7 dpi (J and L) and 9 dpi (K and M).

(N) HE scores at the indicated time points post-infection.

Scale bar: 100 μm (A-M).

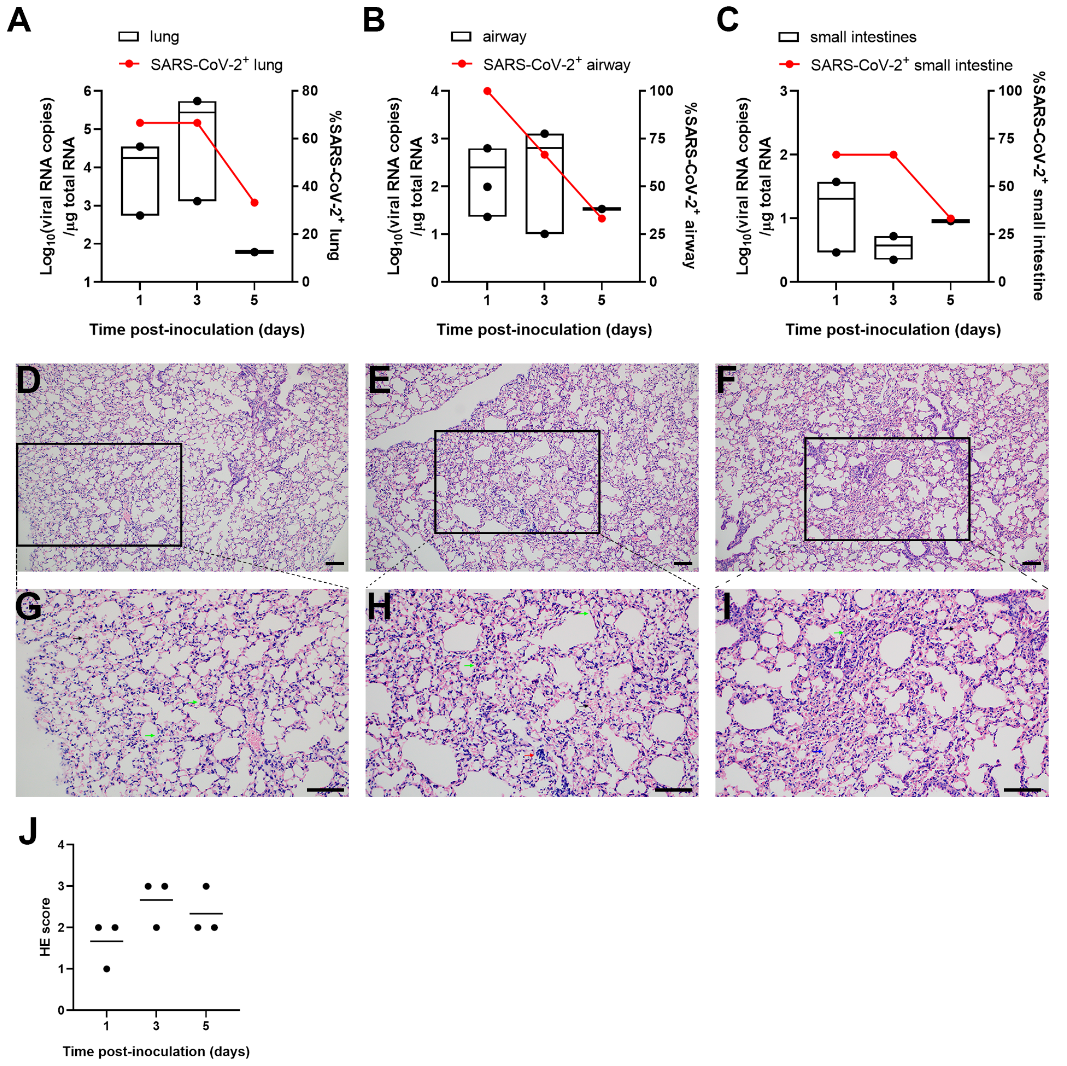

Fig. S4 The virus distribution among tissues and pathological assay in lung tissues of BALB/c hACE2 mice post-infection

(A-C) Different tissues, Lung (A), airway (B), and small intestine (c) were collected after mice sacrifice at the indicated days post-infection. Total tissue RNA was extracted and subjected to qRT-PCR for viral loading assay. The left y-axis showed the viral loading of each tissue, while the right y-axis showed the percentage of tissues containing SARS-CoV-2 in all tissues.

(D-J) Nine SARS-CoV-2-infected BALB/c hACE2 mice were used to assess the lung injury at the indicated time points post-infection. (D and G) Alveolar septal thickening (green arrow) was observed in lung at 1 dpi. (E and H) Alveolar septal thickening (green arrow), hemorrhage (black arrow), and inflammatory cell infiltration (red arrow) were observed in lung at 3 dpi. (F and I) Alveolar septal thickening (green arrow), hyaline membrane formation (blue arrow), and hemorrhage (black arrow) were observed in lung at 5 dpi. (J) HE scores at the indicated time points post-infection. Scale bar: 100 μm (D-I).

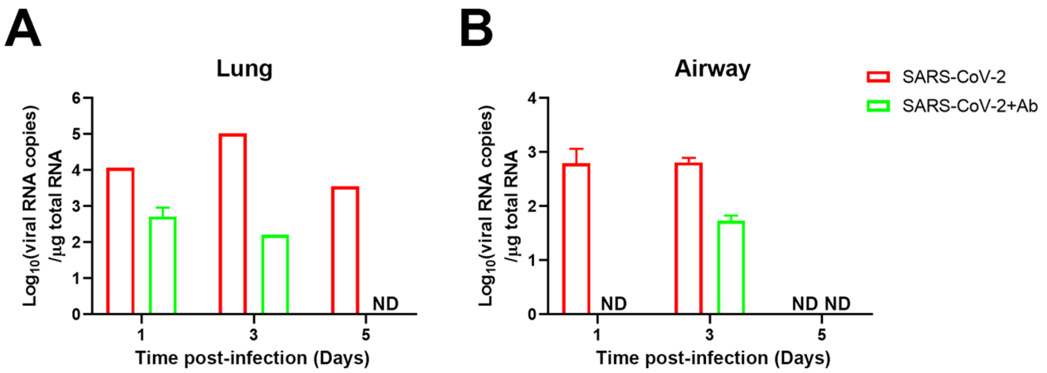

Fig. S5 The Neutralizing antibody suppressed virus replication in lung and airway

ACE2 humanized mice were pretreated with a neutralizing antibody 4 hours before virus challenge. The lung (A) and airway (B) were collected at the indicated days post-infection. Viral loading in the lung and the airway was determined through qRT-PCR.

ND, undetermined. Data are mean ± SD.
