# Supplementary material for: Rapid generation of ACE2 humanized inbred mouse model for COVID-19 with tetraploid complementation
Source: Natl Sci Rev. 2020 Nov 24;8(2):nwaa285. doi: 10.1093/nsr/nwaa285 (PMC7717373; doi:10.1093/nsr/nwaa285)
Supplement: nwaa285_Supplement_File [file nwaa285_supplement_file.zip › MS_perspective_Supplmentary.docx]

**Supplementary**

**Rapid Generation of ACE2 humanized inbred mouse model for COVID-19 with tetraploid complementation**

**EXPERIMENTAL MODEL AND SUBJECT DETAILS**

**Experimental Animals**

This study involved the use of mouse and viral infection in an animal biosafety level 3 (ABSL-3) facility of Kunming Institute of Zoology, Chinese Academy of Sciences. Mice were maintained under specific-pathogen-free (SPF) conditions in ABSL-3 at 23°C and were used when 6- to 10-wk old. All animals were allowed free access to water and diet and provided with a 12 h light/dark cycle. All of the animal experiments were performed following recommendations in the Guide for the Care and Use of Laboratory Animals of Guangzhou Institute of Biomedicine and Health (GIBH), Chinese Academy of Sciences (CAS), and Kunming Institute of Zoology (KIZ), CAS. The Institutional Animal Care and Use in GIBH, CAS, approved the ACE2 humanized mouse generation, while the Institutional Committee for Animal Care and Biosafety at KIZ, CAS, approved works in ABSL-3 (Approval ID: SMKX--tz-20200415-03).

**Cell lines**

C57BL/6 mouse embryonic stem cells were derived from 3.5 d.p.c ICM from C57BL/6 female mice crossing male C57BL/6 mice. ESCs were cultured dependent of feeders in DMEM media with 1000U/mL LIF in DMEM containing 15% fetal calf serum (SL) with or without MEK inhibitor PD0325901 (1 mM) and GSK3 inhibitor CHIR99021 (3 mM), known as 2i (2iL) (Ying et al., 2008).

**Generation of hACE2 mouse ESC**

To construct the targeting vector, cDNAs encoding hACE2 with polyA was inserted into the ATG site of the second exon of mACE2 by homologous recombination, and PGK-Puro flanking with frt served as selection marker. The expression of inserted hACE2 gene was driven by the mouse mACE2 promoter. The specific targeting sgRNA1 (5’-tactgctcagtccctcaccgagg-3’) and sgRNA2 (5’- cttggcattttcctcggtgaggg-3’) along with linear targeting donor were transfected into mouse ESC with electrotransformation.

**Tetraploid Embryos / ES Aggregation**

Super ovulated ICR females are mated with ICR males. E1.5 embryos at the two-cell stage are flushed from the oviducts and placed between the electrodes of a 250 µm gap electrode chamber (BLS Ltd., Budapest, Hungary) containing 0.3 M Mannitol with 0.3% bovine serum albumin (Sigma-Aldrich Inc., St. Louis, MO) and fused with a Cellfusion CF-150/B apparatus (BLS Ltd., Budapest, Hungary) with 0.5 mm Micro fusion slide (BTX-450). An initial electrical field of 3V is applied to the embryos followed by one peak pulses of 50V for 50 µS. The fused tetraploid embryos are cultured to the 4-cell stage for 24 hrs under the same conditions.

ES cells are aggregated and cultured with denuded 4-cell stage mouse tetraploid embryos as reported with a slight modification (Nagy et al., 1993). In detail, Clumps of loosely connected ES cells (15-20 cells in each) from short trypsin-treated Day2 ES cultures are transferred into microdrops of KSOM medium with 10% FCS under mineral oil; Each clump is placed in a depression in the microdrop. Meanwhile, batches of 30-50 embryos are briefly incubated in acidified Tyrode's solution (Brigid, 1986) until dissolution of their zona pellucida. Two embryos are place on one ES clump. All aggregates are assembled in this manner, and cultured overnight at 37C, 5% CO_2_.

After 24 hours of culture, eleven to fifteen embryos are transferred into one uterine horn of a 2.5 dpc pseudopregnant recipient. Mature CD-1 females are used as pseudopregnant foster mothers with a weight of 30+ g.

**RNA Extraction and Real-Time Quantitative PCR**

Total RNA was extracted with a TRIzol-based protocol. For quantitative PCR, cDNA was synthesized with ReverTra Ace (Toyobo) and oligo-dT (Takara), and then analyzed by qPCR with Premix Ex Taq (Takara). Relative expression level of hACE2 and mACE2 was determined using primer pairs hACE2-qF, hACE2-qR and mACE2-qF, mACE2-qR. Amplicons were normalized to expression of GAPDH (glyceraldehyde 3-phosphate dehydrogenase).

**Western Blotting**

Mouse tissues were homogenized in Cell lysis buffer for Western (20 mM Tris-HCl, pH 7.5, containing 150 mM NaCl, 2mM DTT, 50% triglyceride, 100 mM EDTA, 1% SDS, 1% NP40 and 1% Triton X-100) supplemented with Phenylmethanesulfonyl fluoride (PMSF) (Beyotime). Western blots were performed using typical laboratory procedures with the following antibodies: anti-ACE2 (ab108209, Abcam) anti-GAPDH (MAB374, Millipore). Whole-cell extracts were resolved by 12% SDS-PAGE, transferred to PVDF membranes and probed with corresponding antibodies according to the manufacturer’s recommendations (Cell Signaling Technology).

**Histology/Cryosectioning/Immunofluorescence**

Mouse tissue was fixed in 4% PFA overnight at 4°C. The next day the tissue was rinsed 3x in PBS, and cryoprotected in 30% sucrose in PBS overnight at 4°C. Then the tissue was incubated 2-4 hours in a 1:1 mixture of 30% sucrose/PBS and O.C.T. cryoembedding medium (Sakura, cat. no. 4583). Next, groups of 2-4 organoids were transferred from the sucrose/OCT mixture into a cryomold and filled with O.C.T. The embedded tissue was frozen on dry ice, then stored at -80°C until cryostat sectioning. Frozen organoid tissue was sliced into 20 μm sections using a cryostat and collected on superfrost Ultra Plus slides. The sections were dried overnight, and then used for immunofluorescent. 4%PFA was post-fixed directly on slides for 10min at RT followed by washing 3x10 times in PBS. The tissue area was outlined using a hydrophobic PAP pen. Blocking was performed using 5% BSA/0.3% TX100 in PBS with 0.05% sodium azide and incubated 30 minutes at RT. Then the tissue was incubated with anti-ACE2 antibody (ET1611-58, Huabio) or anti-SARS-CoV-2 Nucleocapsid antibody (40143-R019, Sino Biological Inc) 4°C overnight. Following PBS washes, the secondary antibody (A11004, Invitrogen) was applied for 1h and subsequently, DAPI for 2 min. Finally, the coverslips were mounted on slides for observation.

Lungs from virally infected mice were fixed in 4% PFA for at least 7 days, and then were paraffin embedded and cut into 3-μm sections following the standard procedure. The sections were stained with hematoxylin and eosin (H&E), and examined by light microscopy. The pathological score was assessed according the degree of lung tissue lesions including alveolar septal thickening, hemorrhage, inflammatory cells infiltration, and consolidation. The semiquantitative assessment were performed as follows: we scored 0 when no alveolar septal thickening was observed, scored 1 when alveolar septal thickening was very mild and the area of alveolar septal thickening, hemorrhage and inflammatory cells infiltration was less than 10%, scored 2 when alveolar septal thickening was mild and the area of alveolar septal thickening, hemorrhage and inflammatory cells infiltration was 10%-25%, scored 3 when alveolar septal thickening was moderate and the area of alveolar septal thickening, hemorrhage and inflammatory cells infiltration was 25%-50%, scored 4 when alveolar septal thickening was marked and the area of alveolar septal thickening, hemorrhage, inflammatory cells infiltration and consolidation was 50%-75%, and scored 5 when alveolar septal thickening was very marked and the area of alveolar septal thickening, hemorrhage, inflammatory cells infiltration and consolidation was greater than 75%.

**Virus propagation and titration**

The 107 strain of SARS-CoV-2 was kindly provided by Guangdong Provincial Center for Disease Control and Prevention, Guangdong Province of China. The virus was propagated and titrated on Vero E6 cells, which were cultured in Dulbecco’s modified Eagle’s medium (DMEM, Gibco) supplemented with 10% fetal bovine serum.

**Mouse infection and sample collection**

B6 hACE2 mice (n=33) and BALB/c hACE2 mice (n=9) were anesthetized with isoflurane (RWD Life Science, Shenzhen), and then intranasally infected with 2×10^6^ TCID_50_ of SARS-CoV-2 in 30μl of DMEM, respectively. For the mock control (n=9), B6 hACE2 mice were intranasally received 30μl of DMEM. All mice were observed and their body weight and temperature were measured daily until sacrifice.

Fecal samples were freshly collected daily. In brief, once mice defecated 2-3 fecal pellets, fecal pellets were weighed and then dissolved in 1ml of DMEM. At last, fecal samples in medium were store in -80℃ freezer until use.

Tissue sample were collected from sacrificed mice at the indicated time points post-infection. Briefly, mice were deeply anesthetized with isoflurane and sacrificed. Different tissues, including lung, airway, and small intestine, were collected and stored in -80℃ freezer until use.

**RNA extraction and viral loading measurement**

For fecal samples, fecal samples in 1ml of medium were vortexed and then suspension was collected after centrifugation. Two-hundred microliter of fecal suspension was used to extract viral RNA according to the protocol of High Pure Viral RNA Kit (Roche). The extracted RNA was used to measure the nucleoprotein N gene copies of SARS-CoV-2 using THUNDERBIRD^®^ Probe One-step qRT-PCR Kit (Toyobo) as describing in previous studies (Song et al., 2020; Xu et al., 2020). The primer sequences for qRT-PCR were as follows: Forward primer: 5’-GGGGAACTTCTCCTGCTAGAAT-3’; Reverse primer: 5’-CAGACATTTTGCTCTCAAGCTG-3’. The TaqMan probe sequences were 5’-FAM-TTGCTGCTGCTTGACAGATT-TAMRA-3’. The standard samples were purchased from the National Institute of Metrology of China. The viral quantity in fecal sample was expressed as number of copies/g wet weight feces.

Tissue samples were dissolved in 1ml of TRIzol™ Reagent and then subjected to RNA extraction according to the manufacture’s protocol. Similarly, qRT-PCR was also performed to measure viral loading in the tissues. Results were expressed as number of copies/μg tissue total RNA.

**Animal protective assay by neutralizing antibody**

B6 hACE2 mice (n=9) were intravenously administrated with neutralizing antibody (20 mg/kg of body weight), which was kindly provided by Jinghua Yan (Shi et al., 2020), 4 hours before virus challenge. Mice were sacrificed at 1, 3, and 5 days post-infection for tissue (lung and airway) collection.

REFERENCE

Brigid, H. (1986). Manipulating the mouse embryo. A Laboratory Manual.

Nagy, A., Rossant, J., and Joyner, A.L. (1993). Production of completely ES cell-derived fetuses.

Shi, R., Shan, C., Duan, X., Chen, Z., Liu, P., Song, J., Song, T., Bi, X., Han, C., Wu, L.*, et al.* (2020). A human neutralizing antibody targets the receptor binding site of SARS-CoV-2. Nature.

Song, T.Z., Zheng, H.Y., Han, J.B., Jin, L., Yang, X., Liu, F.L., Luo, R.H., Tian, R.R., Cai, H.R., Feng, X.L.*, et al.* (2020). Delayed severe cytokine storm and immune cell infiltration in SARS-CoV-2-infected aged Chinese rhesus macaques. Zool Res *41*, 503-516.

Xu, L., Yu, D.D., Ma, Y.H., Yao, Y.L., Luo, R.H., Feng, X.L., Cai, H.R., Han, J.B., Wang, X.H., Li, M.H.*, et al.* (2020). COVID-19-like symptoms observed in Chinese tree shrews infected with SARS-CoV-2. Zool Res *41*, 517-526.

Ying, Q.L., Wray, J., Nichols, J., Batlle-Morera, L., Doble, B., Woodgett, J., Cohen, P., and Smith, A. (2008). The ground state of embryonic stem cell self-renewal. Nature *453*, 519-523.

**Table 1 The efficiency to generate ACE2 humanized mice by tetraploid complementation (C57BL/6N)**

| ACE2 humanized ESC lines（C57BL/6N） | No. of embryos aggregated | No. of full-term pups (%) | No. of pups survived （%） | No.of live adult mice (%) |
| --- | --- | --- | --- | --- |
| B1 | 216 | 33 (15.3) | 9 (4.2) | 3 (1.4) |
| B2 | 72 | 7 (9.7) | 5 (6.9) | 1 (1.4) |
| B3 | 61 | 0 | 0 | 0 |
| B5 | 267 | 25 (9.4) | 12 (4.5) | 4 (1.5) |
| B10 | 304 | 58 (19.1) | 58 (19.1) | 35 (11.5) |
| B11 | 40 | 4 (10.0) | 4 (10.0) | 2 (5) |
| B12 | 40 | 8 (20.0) | 8 (20.0) | 5 (12.5) |
| B13 | 76 | 12 (15.8) | 11 (14.5) | 4 (5.3) |
| B14 | 1208 | 186 (15.4) | 163 (13.5) | 95 (7.9) |
| B18 | 1231 | 131 (10.6) | 99 (8.0) | 48 (3.9) |
| B20 | 64 | 0 | 0 | 0 |
| B22 | 115 | 17 (14.8) | 15 (13.0) | 11 (9.5) |
| B23 | 53 | 7 (13.2) | 3 (5.7) | 0 |
| B29 | 150 | 0 | 0 | 0 |
| B30 | 72 | 5 (6.9) | 2 (2.8) | 0 |
| B33 | 40 | 3 (7.5) | 3 (7.5) | 0 |
| B39 | 40 | 10 (25.0) | 7 (17.5) | 5 (12.5) |
| B41 | 48 | 16 (33.3) | 15 (31.3) | 12 (25.0) |
| B212 | 40 | 8 (20.0) | 8 (20.0) | 6 (15.0) |
| Total | 4137 | 530 (12.8) | 422 (10.2) | 231 (5.6) |

**Table 2 The efficiency to generate ACE2 humanized mice by tetraploid complementation (BALB/c)**

| ACE2 humanized ESC lines（BALB/c） | No. of embryos aggregated | No. of full-term pups (%) | No. of pups survived （%） | No.of live adult mice (%) |
| --- | --- | --- | --- | --- |
| 4C-1D | 43 | 12 (27.9) | 5 (11.6) | 5 (11.6) |
| 4C-1G | 127 | 16 (12.6) | 9 (7.1) | 9 (7.1) |
| 4C-1H | 986 | 139 (14.1) | 56 (5.7) | 29 2.9) |
| 4C-3A | 41 | 7 (17.1) | 3 (7.3) | 3 (7.3) |
| 4C-3C | 46 | 0 | 0 | 0 |
| 4C-4G | 430 | 39 (9.1) | 31 (7.2) | 21 (7.2) |
| 4C-5C | 46 | 0 | 0 | 0 |
| 4C-5F | 45 | 9 (20.0) | 0 | 0 |
| 4C-6A | 56 | 5 (8.9) | 4 (7.1) | 2 (3.6) |
| 4C-6B | 96 | 1 (1.0) | 0 | 0 |
| 4C-6D | 90 | 5 (5.6) | 3 (3.3) | 3 (3.3) |
| 4C-6F | 78 | 0 | 0 | 0 |
| 4D-1C | 26 | 0 | 0 | 0 |
| 4D-1F | 115 | 11 (9.6) | 11 (9.6) | 11 (9.6) |
| 4D-2B | 38 | 0 | 0 | 0 |
| 4D-3G | 119 | 0 | 0 | 0 |
| 4D-3H | 32 | 0 | 0 | 0 |
| Total | 2414 | 251 (10.4) | 151 (6.3) | 74 (3.1) |

**Table S1. Primer**

| Primer/Probe name | Primer/Probe sequence |
| --- | --- |
| hACE2-qF | GGTCTTCAGTGCTCTCAG |
| hACE2-qR | GCATTCTTGTGGATTATCTGG |
| mACE2-qF | GCAGACAAGAACAAACAGT |
| mACE2-qR | TTTCATCCAATCCTGGCTC |
| SARS-CoV-2-Forward primer | GGGGAACTTCTCCTGCTAGAAT |
| SARS-CoV-2-Reverse primer | CAGACATTTTGCTCTCAAGCTG |
| TaqMan probe | TTGCTGCTGCTTGACAGATT |
